# Association Between Blood Free Fatty Acid Concentrations in Midlife and Cerebral Small Vessel Disease

**DOI:** 10.3390/antiox14091107

**Published:** 2025-09-12

**Authors:** Ryotaro Nukata, Yorito Hattori, Kotaro Noda, Takeshi Yoshimoto, Masafumi Ihara

**Affiliations:** 1 Department of Neurology, National Cerebral and Cardiovascular Center, 6-1 Kishibe-Shimmachi, Suita 564-8565, Osaka, Japan; 2 Department of Preemptive Medicine for Dementia, National Cerebral and Cardiovascular Center, Suita 564-8565, Osaka, Japan; 3 Department of Neurology and Neurological Science, Institute of Science Tokyo Hospital, 1-5-45 Yushima, Bunkyo, Tokyo 113-8510, Japan; 4 Department of Stroke and Cerebrovascular Diseases, University of Tsukuba Hospital, 2-1-1 Amakubo, Tsukuba 305-8576, Ibaraki, Japan

**Keywords:** free fatty acid, cerebral small vessel disease, lacune, cerebral microbleeds

## Abstract

Free fatty acids (FFAs) are a risk factor for recurrent ischemic stroke, primarily via the overproduction of reactive oxygen species. However, the association between FFA concentrations and cerebral small vessel disease (SVD), including lacunes, cerebral microbleeds, and white matter lesions on brain magnetic resonance imaging, remains unclear. This study included 95 patients with acute ischemic stroke (median age: 59 [interquartile range: 49–73] years). The patients were divided into two groups: those aged ≤59 years (midlife patients) and those aged ≥60 years (late-life patients). In the midlife patients, the low serum total FFA concentration was an independent risk factor of lacunes (adjusted odds ratio [aOR]: 0.82, 95% confidence interval [CI]: 0.69–0.96; *p* = 0.013). Among FFA fractions, low serum free C14:0 (aOR: 0.80, 95% CI: 0.66–0.98; *p* = 0.028), and free C18:3n-3 (aOR: 0.93, 95% CI: 0.87–0.99; *p* = 0.015) concentrations were independent risk factors of lacunes in the midlife patients. However, the serum total FFA concentrations did not differ according to the SVD findings in the late-life patients. Therefore, low blood FFA concentrations in midlife can be a novel “nonvascular,” nonatheromatous risk factor of SVD, including the presence of lacunes identified on brain magnetic resonance imaging.

## 1. Introduction

Free fatty acids (FFAs), which are unesterified fatty acids, are present in systemic circulation and produced via the lipolysis of triglycerides in the adipose tissue. FFAs represent an important energy source for organs such as the heart, skeletal muscle, and liver; however, excessive FFA accumulation in non-adipose tissues disrupts multiple metabolic pathways, a process termed lipotoxicity [1]. Elevated blood FFA concentrations are strongly associated with metabolic and cardiocerebrovascular diseases [2,3,4,5,6,7,8,9,10].

In metabolic diseases, FFAs play a crucial role in the development of type 2 diabetes [8] and metabolic dysfunction-associated steatohepatitis (MASH) [9,10]. Regarding the associations between diabetes and FFA, excess FFA levels induce insulin resistance in skeletal muscle, adipose tissue, and liver through elevated mitochondrial and nicotinamide adenine dinucleotide phosphate (NADPH) oxidase-mediated reactive oxygen species (ROS) production [11]. Palmitate, a saturated FFA, directly impairs pancreatic β-cell function by activating NADPH oxidase through Src signaling and inducing endoplasmic reticulum stress, resulting in defective glucose-stimulated insulin secretion and pancreatic β-cell apoptosis [12,13]. Contrarily, antioxidants such as N-acetyl-l-cysteine partially reverse lipid-induced peripheral insulin resistance [14,15]. Regarding MASH, FFAs and oxidative stress are the most important causes. MASH is mainly caused by FFA-induced insulin resistance through increased tumor necrosis factor α expression and fatty acid-induced ROS production, which lead to hepatic fibrosis, inflammation, and cell death [10].

In particular, FFAs are known to contribute to vascular endothelial dysfunction by promoting oxidative stress and inflammation [16,17], and FFA levels are associated with cardiovascular diseases, including coronary artery disease, heart failure, and atrial fibrillation, and atherosclerosis [2,3,4,5,6,7]. Excess FFA levels stimulate ROS production through protein kinase C-dependent NADPH oxidase activation in the presence of high glucose levels in cultured endothelial cells [16]. Palmitate activates nuclear factor-kappa B signaling and subsequently NADPH oxidase through the Toll-like receptor 4 signaling pathway, thereby increasing ROS production in in vivo and in vitro studies [17]. Increased plasma FFA levels are clinically associated with mortality and major adverse cardiovascular events in older patients with coronary artery disease [5], a higher risk of heart failure in older populations [7], and an increased risk of heart failure with preserved ejection fraction in obese patients [18]. Many studies suggest that oxidative stress is a major pathophysiology of atrial fibrillation promoted by atrial remodeling [19]. Elevated levels of FFAs, such as palmitic acid, promote NADPH oxidase 2-dependent ROS production in atrial cardiomyocytes, thereby increasing susceptibility to atrial fibrillation in diet-induced obesity mice [20].

In ischemic stroke, elevated blood FFA concentrations were an independent predictor of recurrent ischemic stroke, poor 90-day functional outcome, and mortality [21]. Further, high serum total FFA concentrations were associated with the severity of carotid artery stenosis in Japanese patients with type 2 diabetes accompanied by hypertension and obesity [22]. As elevated FFA levels increase the risk of atrial fibrillation through increased ROS, FFAs are naturally associated with cardioembolic stroke and its recurrence [23], in which atrial fibrillation is the main cause. In addition, among patients with acute stroke, FFA levels increased with potential sources of cardioembolic stroke irrespective of the presence of atrial fibrillation, suggesting that enhanced thrombogenicity could be the main mechanism of elevated FFA levels in patients with cardioembolic stroke [24].

Cerebral small vessel disease (SVD), commonly associated with aging, is often asymptomatic. However, it carries a high risk of symptomatic lacunar ischemic or hemorrhagic stroke due to cerebral microcirculation impairment [25,26]. SVD contributes to a spectrum of neurological sequelae, including vascular cognitive impairment, neuropsychiatric and mood disorders, mobility issues (such as gait disturbances and parkinsonism), and epilepsy [27,28]. Therefore, it is important to develop preventive strategies against SVD. The neuroradiological findings of SVD on brain magnetic resonance imaging (MRI) are as follows: (1) lacunes, which are caused by a recent small subcortical infarct or hemorrhage; (2) white matter hyperintensities (WMHs), which are associated with impaired cerebrovascular reactivity, and reduced resting cerebral microcirculation; and (3) cerebral microbleeds (CMBs), which reflect underlying arteriolosclerosis—such as blood–brain barrier leakage—or cerebral amyloid angiopathy, and are associated with an increased risk of intracerebral hemorrhage and ischemic stroke [29,30]. Cerebral endothelial cell dysfunction is a key mechanism of SVD [25,31]. Currently, increasing attention has been given to a large “nonvascular,” nonatheromatous etiology in the pathophysiology of SVD, rather than conventional vascular risk factors such as hypertension, diabetes, dyslipidemia, and smoking. This is because controlling conventional vascular risk factors alone has not been established as an effective strategy for preventing SVD. Animal models of chronic cerebral hypoperfusion and white matter injury, mimicking SVD, have shown that oxidative stress is a key mediator of white matter hypoperfusion injury via the expression of NADPH oxidases in various cell types in the brain [32]. Both clinical and experimental studies have shown that FFAs can induce endothelial dysfunction via oxidative stress, inflammation, and decreased nitric oxide production [31,33,34,35,36]. To our knowledge, the relationship between serum FFA concentrations and SVD findings on brain MRI remains unclear, although it is well known that high serum FFA concentrations induce excessive ROS production, which is a key mechanism of SVD. We hypothesized that serum FFA concentrations are correlated with the severity of SVD on brain MRI. Thus, the current study aimed to investigate the association between serum FFA concentrations and SVD findings and the severity of SVD on brain MRI.

## 2. Materials and Methods

### 2.1. Study Design

This cross-sectional observational study was conducted at the National Cerebral Cardiovascular Center (NCVC) in Japan. It was carried out in accordance with the Declaration of Helsinki and was approved by the Research Ethics Committee of NCVC (approval number: M29-003 and R20113). All patients were admitted to the hospital for ischemic stroke between August 2012 and May 2017 and provided written informed consent for the NCVC Biobank to measure serum FFA concentrations. Ischemic stroke was diagnosed via neurological examinations performed by certified vascular neurologists at our institution and brain computed tomography scan or MRI.

### 2.2. Measurement of FFA Concentrations

Fasting blood samples were collected during hospitalization, and serum total FFA and each FFA fraction level were measured using gas chromatography-mass spectrometry (Shimadzu Techno-Research, Kyoto, Japan). The FFA fractions included free C12:0, C14:0, C16:0, C16:1, C18:0, C18:1n9c, C18:2n6c, C18:3n3, C20:1n9, C20:2n6, C20:3n6, C20:4n6, C20:5n3, and C22:6n3.

### 2.3. Clinical Characteristics of the Participants

Ischemic stroke was diagnosed based on the presence of cardioembolic stroke, large-artery atherosclerosis, small-vessel occlusion, and stroke of other determined etiologies based on Trial of Org 10172 in Acute Stroke Treatment [37]. The following variables were obtained from the electronic medical records: age, sex, smoking, and body mass index (kg/m^2^) upon admission. Patients’ serum total bilirubin, aspartate aminotransferase, and alanine aminotransferase levels were extracted from medical records to check liver function, as serum FFA concentrations might be influenced by liver function. Comorbidities were diagnosed according to the following criteria: hypertension, defined as a systolic blood pressure ≥ 140 mmHg, a diastolic blood pressure ≥ 90 mmHg, or a history of antihypertensive medication use. Diabetes mellitus was defined as a fasting plasma glucose level ≥ 126 mg/dL, a hemoglobin A1c level ≥ 6.5%, or a history of antidiabetic drugs or insulin use. Dyslipidemia was defined as a low-density lipoprotein cholesterol level ≥ 140 mg/dL, a high-density lipoprotein cholesterol level ≤ 40 mg/dL, a triglyceride level ≥ 150 mg/dL, or a history of lipid-lowering drug use.

### 2.4. Brain MRI Findings

During hospitalization, brain MRI examinations were performed on all patients using 3.0-Tesla scanners (Verio, Vida or Spectra: Siemens, Erlangen, Germany; Premier: GE, Boston, MA, USA; Elition: Philips, Amsterdam, The Netherlands) at the NCVC. SVD findings, such as lacunes, CMBs, and WMHs, including periventricular hyperintensities (PVHs), and deep and subcortical white matter hyperintensities (DSWMHs) were assessed on brain MRI. Lacune was defined as an ovoid, subcortical, fluid-filled cavity, with a diameter of 3–15 mm on fluid-attenuated inversion recovery imaging [10]. The PVH and DSWMH were graded (0–3) based on the previously proposed Fazekas scale [38]. CMBs on T2*-weighted images were assessed and classified as either deep or lobar [10] based on the Microbleed Anatomical Rating Scale [39]. All SVD findings were independently evaluated by two well-trained neurologists (R.N. and K.N.).

### 2.5. Statistical Analysis

Continuous variables were presented as median and interquartile range (IQR), and categorical variables were expressed as frequencies with percentages. The chi-square test was used to assess differences in categorical variables. Meanwhile, the Mann–Whitney U test or the *t*-test was used for ordinal and continuous variables, as appropriate. Multivariate logistic regression analysis adjusted for sex, hypertension, diabetes mellitus, and dyslipidemia was conducted to identify the independent risk factors of SVD findings on brain MRI. Subgroup analysis was conducted according to sex. In this analysis, multivariate logistic regression was performed with adjustment for hypertension, diabetes mellitus, and dyslipidemia. All reported *p*-values were two-tailed, and *p*-values of <0.05 indicated statistically significant differences. All statistical analyses were performed using the Statistical Package for the Social Sciences software version 29 (IBM Inc., Armonk, NY, USA), GraphPad PRISM version 8.4.3. (GraphPad Software, Boston, MA, USA), and R version 4.4.3 (Vienna, Australia).

## 3. Results

### 3.1. Baseline Characteristics of the Patients

Of the patients who provided written informed consent for the NCVC Biobank, 95 underwent measurements for serum FFA concentrations. The median age of the patients was 59 (IQR: 49–73) years. As SVD is frequently associated with aging, as mentioned in the Introduction section, all patients were divided into two groups: patients aged ≤59 (IQR: 42.5–55) years (midlife patients) and those aged ≥60 (IQR: 67–77) years (late-life patients). The midlife patients had a significantly higher body mass index (24.4 vs. 22.2; *p* = 0.003), lower aspartate aminotransferase levels (19.5 U/L vs. 23 U/L; *p* = 0.034), a lower prevalence of cardioembolic stroke (0.0% vs. 14.9%; *p* = 0.005) and small-vessel occlusion (12.5% vs. 36.2%; *p* = 0.007), and a higher prevalence of stroke of other determined etiologies (45.8% vs. 14.9%; *p* = 0.001) than the late-life patients (Table 1).

### 3.2. Inverse Association Between the Total Serum FFA Concentrations and SVD in the Midlife Patients

**Figure 1:**
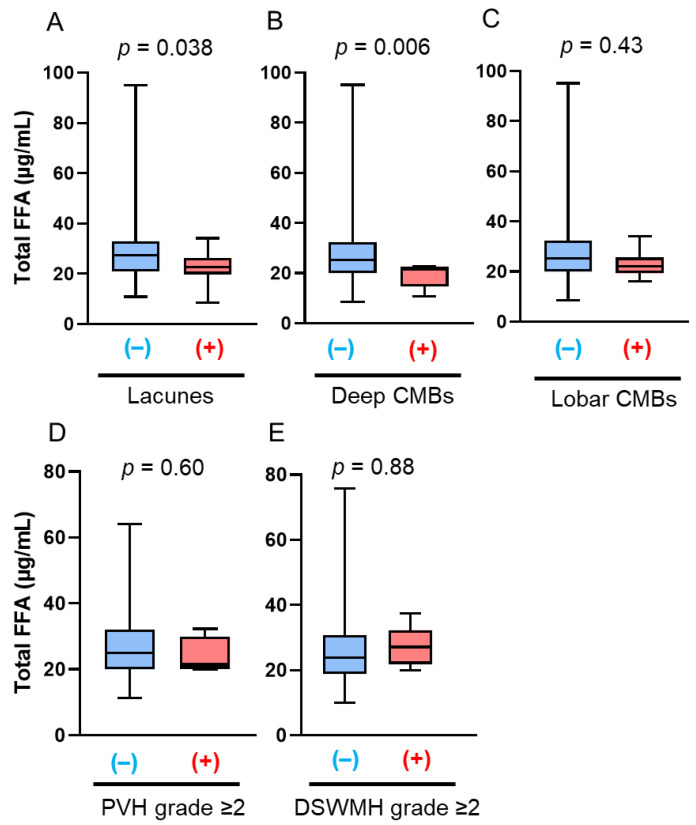
Comparison of total free fatty acid (FFA) concentrations based on the presence of magnetic resonance imaging findings of cerebral small vessel disease (SVD) in midlife patients. The bar graphs show the total FFA concentrations according to the presence of the following SVD findings: (**A**) lacunes, (**B**) deep cerebral microbleeds (CMBs); (**C**) lobar CMBs; (**D**) periventricular hyperintensity (PVH) grade > 2; and (**E**) deep subcortical white matter hyperintensity (DSWMH) grade > 2. (+) indicates presence of each finding; (–) indicates absence of each finding.

**Figure 2:**
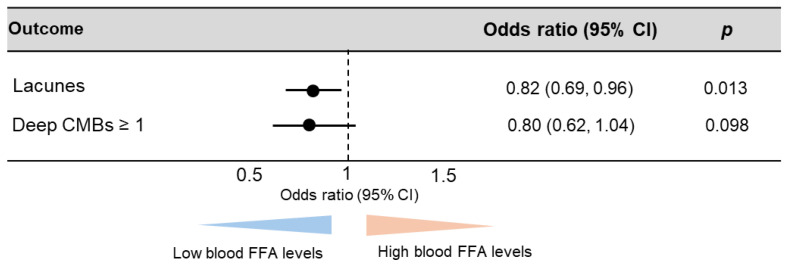
Association between total free fatty acid (FFA) concentrations and the presence of lacunes and deep cerebral microbleeds (CMBs) in midlife patients. Abbreviation: CI, confidence interval. The multivariate logistic regression analyses were adjusted for sex, hypertension, diabetes mellitus, and dyslipidemia.

**Figure 3:**
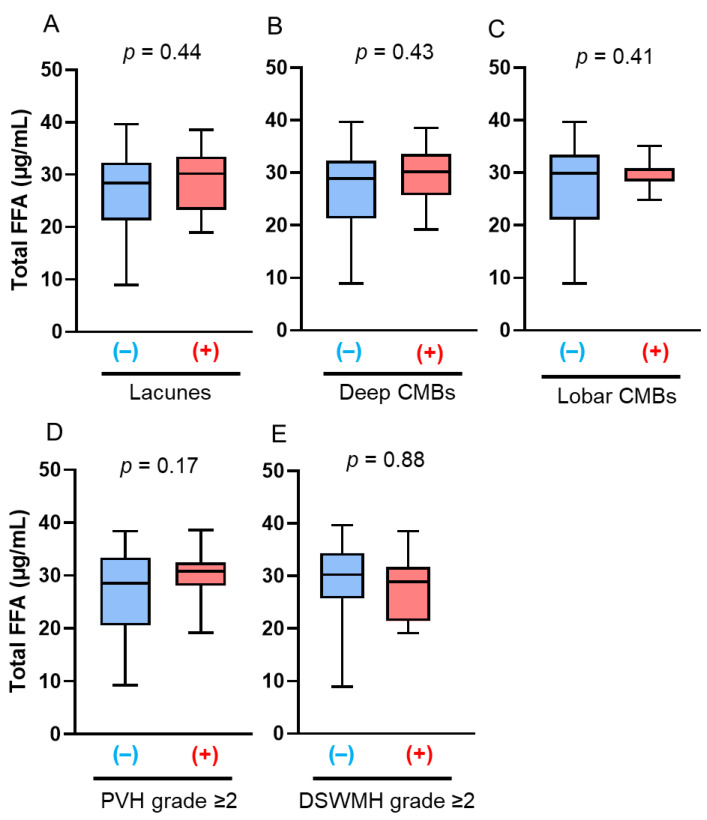
Comparison of total free fatty acid (FFA) concentrations according to the presence of brain magnetic resonance imaging findings of cerebral small vessel disease (SVD) in late-life patients. The bar graphs show the total FFA concentrations according to the presence of the following SVD findings: (**A**) lacunes, (**B**) deep cerebral microbleeds (CMBs); (**C**) lobar CMBs; (**D**) periventricular hyperintensity (PVH) grade > 2; and (**E**) deep subcortical white matter hyperintensity (DSWMH) grade > 2. (+) indicates presence of each finding; (–) indicates absence of each finding.

The late-life patients had a higher prevalence of SVD findings on brain MRI, including lacunes, CMBs, and WMHs, than the midlife patients, which is consistent with previous reports (Table 2). In the midlife patients, those with lacunes (22.6 µg/mL vs. 27.3 µg/mL; *p* = 0.038) and deep CMBs (21.5 µg/mL vs. 25.2 µg/mL; *p* = 0.006) had significantly lower serum total FFA concentrations than those without (Figure 1A,B). PVH grade ≥ 2, DSWMH grade ≥ 2, or lobar CMBs were not associated with altered serum total FFA concentrations (Figure 1C–E). Multivariate logistic regression analyses showed that the serum total FFA concentrations were inversely associated with the presence of lacunes (adjusted odds ratio [aOR]: 0.82, 95% confidence interval [CI]: 0.69–0.96; *p* = 0.013), and were more likely to be inversely associated with deep CMBs (aOR: 0.80, 95% CI: 0.62–1.04; *p* = 0.098) (Figure 2). By contrast, in late-life patients, those with lacunes (*p* = 0.44), deep CMBs (*p* = 0.43), or PVH of grade ≥ 2 (*p* = 0.17) tended to exhibit higher total FFA concentrations; however, these differences were not statistically significant (Figure 3). There were no significant differences in liver function test results—including total bilirubin, aspartate aminotransferase, and alanine aminotransferase—between midlife patients with and without lacunes or deep CMBs (Table 3).

**Figure 4:**
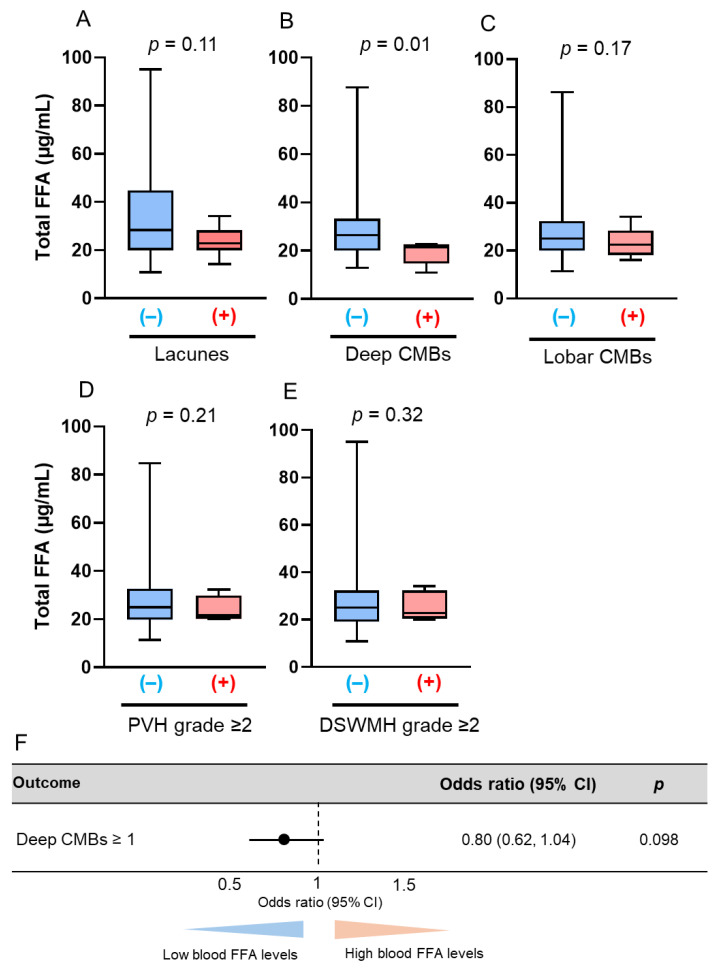
Comparison of total free fatty acid (FFA) concentrations according to the presence of brain magnetic resonance imaging findings of cerebral small vessel disease (SVD) in midlife men. The bar graphs show the total FFA concentrations according to the presence of the following SVD findings: (**A**) lacunes, (**B**) deep cerebral microbleeds (CMBs); (**C**) lobar CMBs; (**D**) periventricular hyperintensity (PVH) grade > 2; and (**E**) deep subcortical white matter hyperintensity (DSWMH) grade > 2. (+) indicates presence of each finding; (–) indicates absence of each finding. (**F**) Multivariate logistic regression analysis was adjusted for hypertension, diabetes mellitus, and dyslipidemia. Abbreviation: CI, confidence interval.

**Figure 5:**
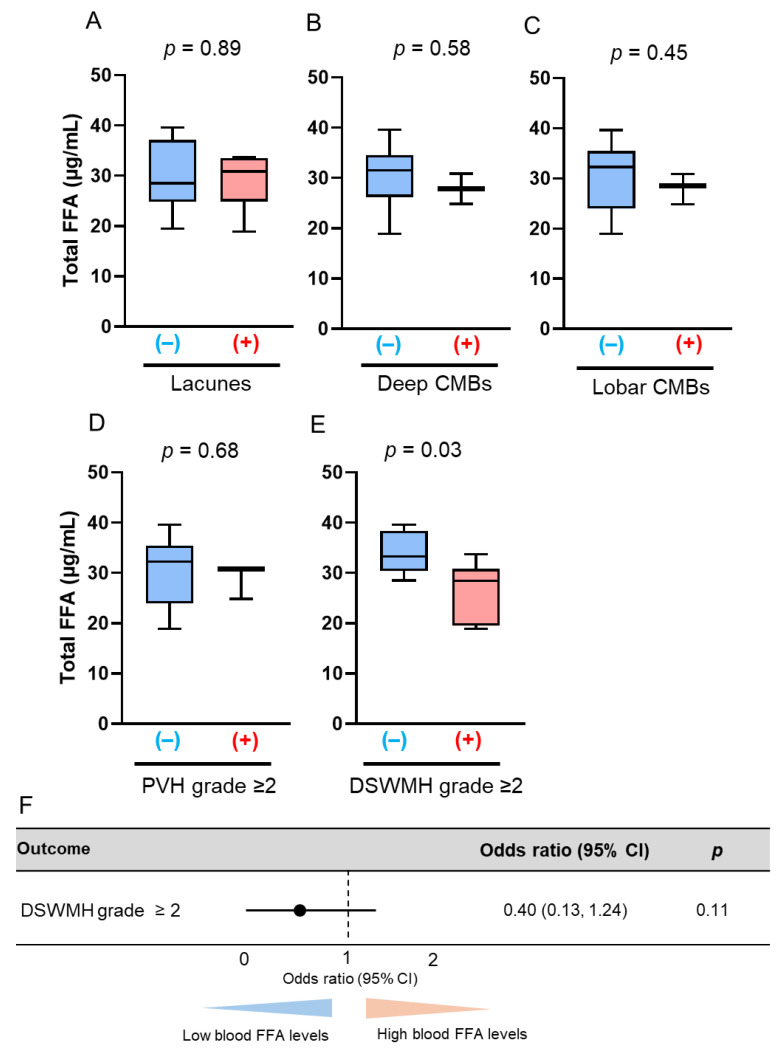
Comparison of total free fatty acid (FFA) concentrations according to the presence of brain magnetic resonance imaging findings of cerebral small vessel disease (SVD) in late-life women. The bar graphs show the total FFA concentrations according to the presence of the following SVD findings: (**A**) lacunes, (**B**) deep cerebral microbleeds (CMBs); (**C**) lobar CMBs; (**D**) periventricular hyperintensity (PVH) grade > 2; and (**E**) deep subcortical white matter hyperintensity (DSWMH) grade > 2. (+) indicates presence of each finding; (–) indicates absence of each finding. (**F**) Multivariate logistic regression analysis was adjusted for hypertension, diabetes mellitus, and dyslipidemia. Abbreviation: CI, confidence interval.

**Figure 6:**
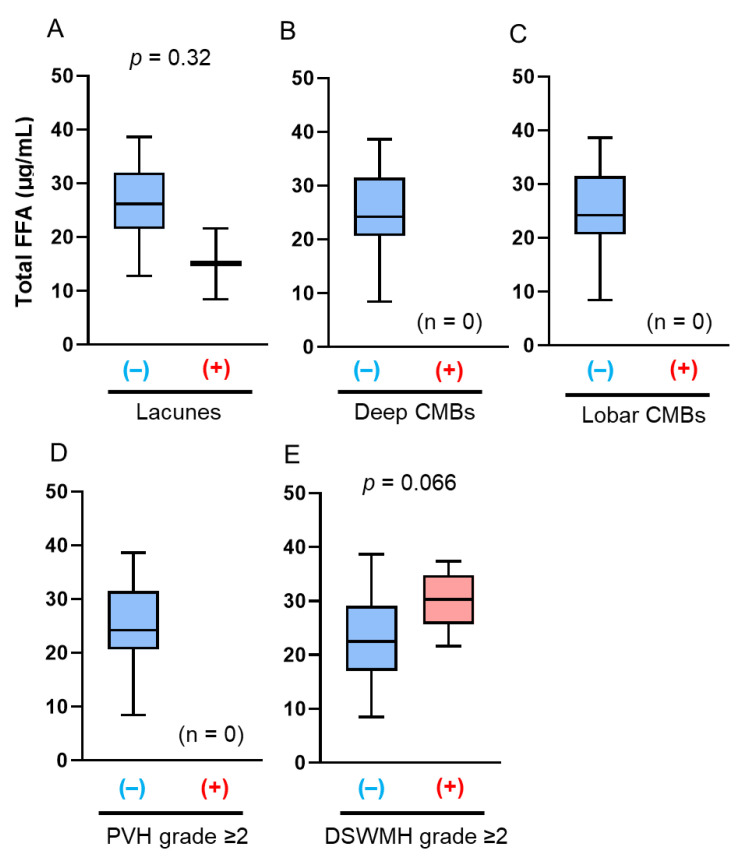
Comparison of total free fatty acid (FFA) concentrations according to the presence of brain magnetic resonance imaging findings of cerebral small vessel disease (SVD) in midlife women. The bar graphs show the total FFA concentrations according to the presence of the following SVD findings: (**A**) lacunes, (**B**) deep cerebral microbleeds (CMBs); (**C**) lobar CMBs; (**D**) periventricular hyperintensity (PVH) grade > 2; and (**E**) deep subcortical white matter hyperintensity (DSWMH) grade > 2. (+) indicates presence of each finding; (–) indicates absence of each finding.

**Figure 7:**
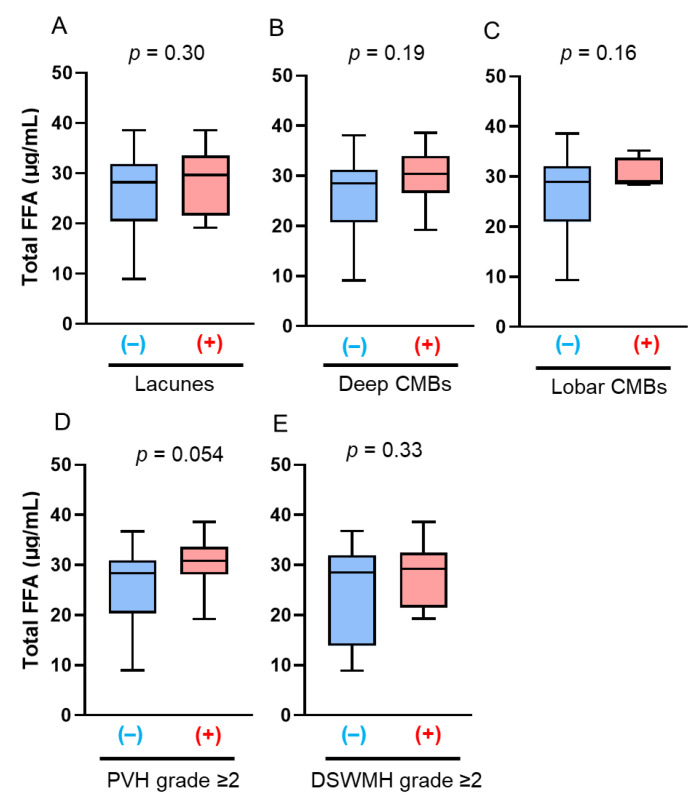
Comparison of total free fatty acid (FFA) concentrations according to the presence of brain magnetic resonance imaging findings of cerebral small vessel disease (SVD) in late-life men. The bar graphs show the total FFA concentrations according to the presence of the following SVD findings: (**A**) lacunes, (**B**) deep cerebral microbleeds (CMBs); (**C**) lobar CMBs; (**D**) periventricular hyperintensity (PVH) grade > 2; and (**E**) deep subcortical white matter hyperintensity (DSWMH) grade > 2. (+) indicates presence of each finding; (–) indicates absence of each finding.

Next, serum total FFA concentrations were analyzed according to sex and age. We separated the patients into four groups: midlife men (*n* = 30), midlife women (*n* = 18), late-life men (*n* = 35), and late-life women (*n* = 12). Midlife men with deep CMBs had significantly lower serum total FFA concentrations than those without deep CMBs (21.45 mg/dL vs. 25.34 mg/dL; *p* = 0.01, Figure 4B), and late-life women with DSWMH had significantly lower serum total FFA concentrations than those without DSWMH (28.43 mg/dL vs. 33.29 mg/dL; *p* = 0.01, Figure 5E). However, serum total FFA concentrations were not associated with deep CMBs in midlife patients (aOR: 0.80, 95% CI: 0.62–1.04; *p* = 0.098, Figure 4F) or DSWMH in late-life patients (aOR: 0.40, 95% CI: 0.13–1.24; *p* = 0.11, Figure 5F) in multivariate logistic regression analyses. No differences in other neuroradiological findings of SVD were observed among the groups (Figure 4A,C–E, Figure 5A–D, Figure 6 and Figure 7).

**Figure 8:**
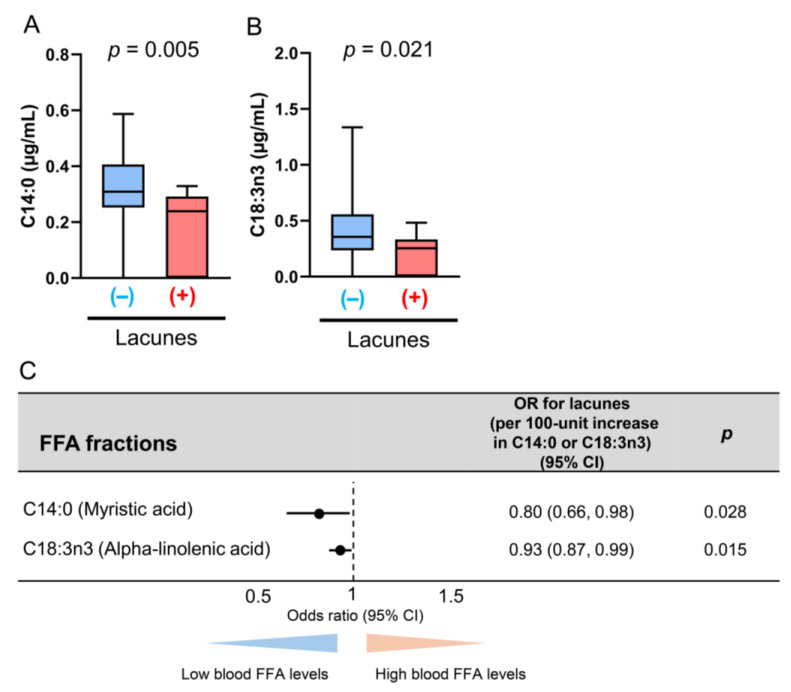
Associations between the free C14:0 and C18:3n3 concentrations and lacunes in the midlife patients. The bar graphs show the free C14:0 (**A**) and C18:3n3 (**B**) concentrations according to the presence of lacunes. (+) indicates presence of lacunes; (–) indicates absence of lacunes. The multivariate logistic regression analyses were adjusted for sex, hypertension, diabetes mellitus, and dyslipidemia (**C**). Abbreviations: CI, confidence interval; FFA, free fatty acid.

Among the FFA fractions, the serum free C14:0 (*p* = 0.005) and C18:3n3 (*p* = 0.021) concentrations were significantly lower in the midlife patients with lacunes (Figure 8A,B). Based on the multivariate logistic regression analyses, the serum free C14:0 level (aOR for a 100-unit increase in the free C14:0 level: 0.80, 95% CI: 0.66–0.98; *p* = 0.028) and the free C18:3n-3 concentrations (aOR for a 100-unit increase in the free C18:3n3 level: 0.93, 95% CI: 0.87–0.99; *p* = 0.015) were inversely associated with the presence of lacunes in the midlife patients (Figure 8C). The serum concentrations of the other FFA fractions were not associated with lacunes (Table 4).

## 4. Discussion

The main and unexpected finding of this study was that the low serum total FFA concentrations were significantly associated with the presence of lacunes in the midlife patients. However, the late-life patients did not present with such an association. Our results indicated that low serum total FFA concentrations during the midlife period contribute to the development of SVD.

Thus far, only high blood FFA concentrations have been considered a strong risk factor of metabolic syndrome, such as type 2 diabetes [8], ischemic heart disease [3,4], and ischemic stroke [21]. However, a recent prospective longitudinal study that included a Chinese cohort has reported that low and high blood FFA concentrations are associated with a higher risk of mortality and ischemic events in coronary artery disease in patients with type 2 diabetes. According to the result, there is a non-liner U-shaped curve relationship between blood FFA concentrations and mortality and ischemic events [40]. Thus, low blood FFA concentrations can also contribute to the development of ischemic disease. There are two plausible explanations for the findings of our study. First, midlife vascular health generally influences SVD burden. Whitehall II Imaging Study [41,42] and Framingham Heart Study [42] have revealed that midlife or cumulative mid-to-late-life hypertension has a major role in SVD findings on brain MRI. Similarly, our study showed that low blood FFA concentration in midlife was associated with the presence of lacunes. We hypothesized that low blood FFA concentrations in midlife did not act as a preconditioning factor for preventing lacunes. In contrast, high blood FFA concentration may be a preconditioning factor via elevated oxidative stress levels, which activates autophagy and antioxidants such as manganese superoxide dismutase and nuclear factor erythroid 2-related factor 2 [43,44,45,46,47]. Second, the apparent contradictory findings in this study suggest another explanation that blood FFA concentrations have a biphasic course from the early to late phase of SVD. Several analogous examples support this concept. Amyloid-β_1–42_ levels in the cerebrospinal fluid generally decrease during the symptomatic stage of Alzheimer’s disease (AD). However, an increase in cerebrospinal fluid amyloid-β_1–42_ levels was found in the preclinical phase in mouse AD models [48] and patients with early AD [49]. The estimated glomerular filtration rate of patients with diabetic nephropathy shows hyperfiltration in the initial phase, followed by a progressive decrease in glomerular filtration rate values that significantly exceed what is expected for age. This initial phase of hyperfiltration may be a compensatory mechanism that counteracts endothelial dysfunction, arterial stiffness, and chronic low-grade renal inflammation. The right ventricular outflow tract acceleration time (RVOT-ACT), which is evaluated with echocardiography, is initially prolonged in the asymptomatic phase of *RNF213* p.R4810K-related pulmonary hypertension in our previous study [50]. However, symptomatic pulmonary hypertension generally is associated with a shortened RVOT-ACT, thereby reflecting an increased pulmonary arterial pressure. Thus, several diseases indicate the biphasic nature of disease markers.

Contrarily, the serum total FFA concentration was not significantly different between late-life patients with and without SVD findings on brain MRI in this study. Several factors may account for these findings. First, SVD primarily develops as a consequence of long-term cumulative exposures, such as midlife hypertension [41,42] and aging, whereas late life represents a relatively shorter period of exposure; thus, the influence of late-life factors may be limited. Second, the associations between each risk factor and SVD may be attenuated in older age, as aging itself is the strongest risk factor, and elderly individuals typically present with multiple conventional vascular risk factors that collectively contribute to SVD development. Nonetheless, patients with lacunes, deep CMBs, or PVH grade ≥ 2 tended to exhibit higher total FFA concentrations in this study (Figure 3), suggesting a biphasic course of FFA levels from the early to late stages of SVD.

Our study specifically showed that the serum C14:0 and C18:3n3 concentrations were significantly lower in the midlife patients presenting with lacunes. Focusing on oxidative stress, C14:0 (myristic acid), a free fatty acid highly abundant in copra/palmist oils, is a predictor of nonalcoholic steatohepatitis, and it stimulates ceramide synthesis [51,52,53]. Ceramide synthesis exacerbates the production of reactive oxidative species via endoplasmic reticulum stress and mitochondrial dysfunction [53]. C18:3n3 (alpha-linolenic acid), an omega-3 polyunsaturated fatty acid, stimulates the production of oxidative stress by reducing the release of nitric oxide with a simultaneous increase in lipid peroxidation [54], and the depletion of vitamin E, which is a potent antioxidant in experimental studies [55]. Therefore, higher serum concentrations of free myristic acid and alpha-linolenic acid in midlife may contribute to the prevention of lacunes via a preconditioning mechanism, whereas lower FFA concentrations in midlife might not confer such preconditioning.

Lacunes and deep CMBs as neuroradiological findings on brain MRI are basically asymptomatic lesions. However, they are recognized as predictors of ischemic stroke and intracerebral hemorrhage [56,57,58]. Therefore, preventing the development of lacunes and deep CMBs could reduce the risk of future symptomatic stroke. Regarding clinical implications, further large-scale observational studies are needed to determine the optimal cutoff value of serum total FFA concentrations in midlife for predicting the risk of SVD. Midlife patients with FFA concentrations below this cutoff value should receive more intensive control of vascular risk factors to prevent the development and progression of SVD.

Despite its strengths, the current study has several limitations. First, this was a cross-sectional observational study. Thus, a longitudinal prospective study should be performed to establish a causal association between blood FFA concentrations and the development or exacerbation of SVD. Second, brain MRI was performed during the acute phase of ischemic stroke. Therefore, there could be mixed asymptomatic SVD findings with acute findings of ischemic stroke on brain MRI. Third, we did not assess insulin resistance and collect data on dietary patterns, which could affect blood FFA concentrations. For instance, some patients might have been taking fish oil supplements, which can increase the levels of certain fatty acids, prior to admission. Fourth, this observational study was conducted at a single institution in Japan, and the generalizability of the findings to other populations and ethnicities is limited. Finally, the sample size of this study might have been insufficient because the measurement of serum FFAs levels is costly (USD 355 per sample). Therefore, this study should be considered exploratory. To enhance statistical power and validity, future studies should include a larger cohort.

## 5. Conclusions

Low blood FFA concentrations in midlife could be a novel “nonvascular,” nonatheromatous risk factor of SVD, including the presence of lacunes detected on brain MRI. Nevertheless, more studies should be performed to validate these findings and the role of FFA on SVD.

## Figures and Tables

**Table 1:** Baseline characteristics of the study population.

	All Patients (*n* = 95)	Midlife Patients (*n* = 48)	Late-Life Patients (*n* = 47)	*p*-Value
Age [years]	59 (49–73)	49.5 (42.5–55)	73 (67–77)	—
Female sex	30 (31.6)	18 (37.5)	12 (25.5)	0.21
Smoking	54 (56.8)	26 (54.2)	28 (59.6)	0.60
Hypertension	72 (75.8)	36 (75.0)	36 (76.6)	0.86
Diabetes	29 (30.5)	15 (31.3)	14 (29.8)	0.88
Dyslipidemia	62 (65.3)	30 (62.5)	32 (68.1)	0.57
Body mass index [kg/m^2^]	23.3 (21.0–26.4)	24.4 (22.7–27.0)	22.2 (19.9–25.3)	0.003
Liver function				
- Total bilirubin [mg/dL]	0.6 (0.43–0.7)	0.5 (0.4–0.63)	0.6 (0.5–0.7)	0.13
- Aspartate aminotransferase [U/L]	21 (16–27.5)	19.5 (16–23)	23 (18–29)	0.034
- Alanine aminotransferase [U/L]	16 (13–22)	17.5 (14–23.25)	16 (12–19.5)	0.24
Subtypes of ischemic stroke				
- Cardioembolic stroke	7 (7.4)	0 (0.0)	7 (14.9)	0.005
- Large-artery atherosclerosis	36 (37.8)	20 (41.7)	16 (34.0)	0.44
- Small-vessel occlusion	23 (24.2)	6 (12.5)	17 (36.2)	0.007
- Stroke of other determined etiologies	29 (30.5)	22 (45.8)	7 (14.9)	0.001

Continuous variables are expressed as median with interquartile range, and categorical variables are presented as frequencies with percentages.

**Table 2:** Prevalence of brain magnetic resonance imaging findings of cerebral small vessel disease and serum total free fatty acid concentrations.

	All Patients (*n* = 95)	Midlife Patients (*n* = 48)	Late-Life Patients (*n* = 47)
Lacunes	42 (44.2)	18 (37.5)	24 (51.1)
Deep CMBs	18 (20.0)	6 (13.0)	12 (27.3)
Lobar CMBs	13 (14.4)	6 (13.0)	7 (15.9)
PVH grade > 2	22 (23.2)	4 (8.3)	18 (38.3)
DSWMH grade > 2	49 (51.6)	16 (33.3)	33 (70.2)
Total FFA levels [µg/mL]	28.4 (21.0–32.3)	24.7 (20.0–31.7)	29.5 (23.2–32.8)

Continuous variables were presented as median with interquartile range, and categorical variables were expressed as frequencies with percentages. Abbreviations: CMBs, cerebral microbleeds; PVH, periventricular hyperintensities; DSWMH, deep subcortical white matter hyperintensities; FFA, free fatty acid.

**Table 3:** Comparison of liver function indices according to the presence of small vessel disease (SVD) findings on brain magnetic resonance imaging (MRI) in midlife patients.

	All Patients	SVD Findings on Brain MRI (−)	SVD Findings on Brain MRI (+)	*p*-Value
Lacunes				
Number	*n* = 48	*n* = 30	*n* = 18	
Total bilirubin [mg/dL]	0.50 (0.40–0.65)	0.50 (0.40–0.60)	0.60 (0.50–0.70)	0.20
Aspartate aminotransferase [U/L]	20 (16–23)	20 (16–23)	20 (16–26)	0.72
Alanine aminotransferase [U/L]	18 (14–24)	16 (14–23)	20 (14–27)	0.41
Deep CMBs				
Number	*n* = 46	*n* = 40	*n* = 6	
Total bilirubin [mg/dL]	0.50 (0.40–0.70)	0.50 (0.40–0.70)	0.55 (0.40–0.70)	>0.99
Aspartate aminotransferase [U/L]	19 (16–23)	19 (16–25)	20 (19–21)	>0.99
Alanine aminotransferase [U/L]	17 (14–24)	16 (14–24)	18 (14–21)	>0.99

Values are expressed as the median and interquartile range. Abbreviation: CMBs, cerebral microbleeds.

**Table 4:** Comparison of free fatty acid (FFA) fraction concentrations based on the presence of lacunes in the midlife patients.

FFA Fraction [µg/mL]	Patients Without Lacunes (*n* = 30)	Patients with Lacunes (*n* = 18)	*p* Value
C12:0	0 (0–0)	0 (0–0)	0.37
C16:0	7.14 (6.21–8.63)	6.55 (5.99–8.39)	0.40
C16:1	0.63 (0.33–1.05)	0.42 (0.3–0.6)	0.072
C18:0	3.08 (2.54–4.23)	2.77 (2.56–3.87)	0.61
C18:1n9c	8.5 (6.28–11.26)	7.06 (4.66–8.71)	0.083
C18:2n6c	3.64 (2.73–5.35)	3.25 (2.19–3.57)	0.15
C20:1n9	0 (0–0)	0 (0–0)	0.28
C20:2n6	0 (0–0)	0 (0–0)	0.46
C20:3n6	0 (0–0)	0 (0–0)	0.59
C20:4n6	0.62 (0.41–0.95)	0.42 (0.33–0.75)	0.10
C20:5n3	0 (0–0.15)	0 (0–0)	0.64
C22:6n3	0.65 (0.51–1.11)	0.44 (0.36–0.85)	0.14

Values are expressed as median (interquartile range).

## Data Availability

The data supporting the findings of this study are available upon request from the corresponding author. The data are not publicly available due to privacy or ethical restrictions.

## Abbreviations

The following abbreviations are used in this manuscript:

AD	Alzheimer’s disease
aOR	Adjusted odds ratio
CI	Confidence interval
CMBs	Cerebral microbleeds
DSWMH	Deep subcortical white matter hyperintensity
FFA	Free fatty acid
IQR	Interquartile range
MASH	Metabolic dysfunction-associated steatohepatitis
MRI	Magnetic resonance imaging
NADPH	Nicotinamide adenine dinucleotide phosphate
NCVC	National Cerebral and Cardiovascular Center
PVH	Periventricular hyperintensity
ROS	Reactive oxygen species
RVOT-ACT	Right ventricular outflow tract acceleration time
SVD	Cerebral small vessel disease
WMHs	White matter hyperintensities
